# Nano biofertilizer: a sustainable blueprint for restoring soil fertility under stress conditions

**DOI:** 10.3389/fpls.2026.1894680

**Published:** 2026-10-21

**Authors:** Ravi Kushwaha, Manish Dev, Amit Kumar, Shraddha Shukla, Astha Srivastava, Shashi Kant Shukla

**Affiliations:** 1 Anupam Rural Laboratory, Centre of Science and Society, Institute of Interdisciplinary Studies (IIDS), University of Allahabad, Prayagraj, India; 2 K. Banerji Centre of Atmospheric and Oceanic Studies, University of Allahbad, Prayagraj, India

**Keywords:** abiotic stress, nano-biofertilizers, nano-fertilizers, organic farming, plant growth-promoting rhizobacteria

## Abstract

Agricultural productivity has persistently diminished due to various influences including anthropogenic activity, climate change and abiotic stress, raising alarms regarding food security and chronic hunger-like situations affecting millions of people worldwide. Abiotic stress consists of drought, salinity, extreme weather and heavy metals which damages the soil quality, microbial diversity and crop yields. Chemical fertilizers emerge in the scene to cope up with abovesaid circumstances but reduces microbial biodiversity, soil health and pose a threat to humans at the same time. Natural farming offers a conventional and eco-friendly pathway to achieve sustainable agriculture and Sustainable Development Goal 2 (Zero Hunger), which includes beneficial soil microbes such as Plant growth promoting rhizobacteria (Biofertilizer/PGPR), but their low efficacy requires an innovative approach. Meanwhile, nanotechnology has been listed as a promising tool to enhance agricultural output and stress resilience but also raises concerns over damaging soil health, accumulation and potential health risks. To overcome these issues, an inventive technology including nanoparticles (NPs) and biofertilizers tied to formulate Nano-biofertilizers (NBFs), a potential solution to modern agriculture. NBFs are very specific and enhances the growth of plants by controlled and targeted delivery at molecular level, highly absorbent due to their size and concentration and upsurge microbial colonization. They are well known agents of climate resilience and escape microbes and plants from abiotic stress conditions. NBFs efficiently and progressively acts in the cellular level of plants in order to promote them. The current review tries to draw a blueprint about concept, aspects and various prospects of NBFs in the light abiotic stress conditions.

## Graphical Abstract

**Figure:**
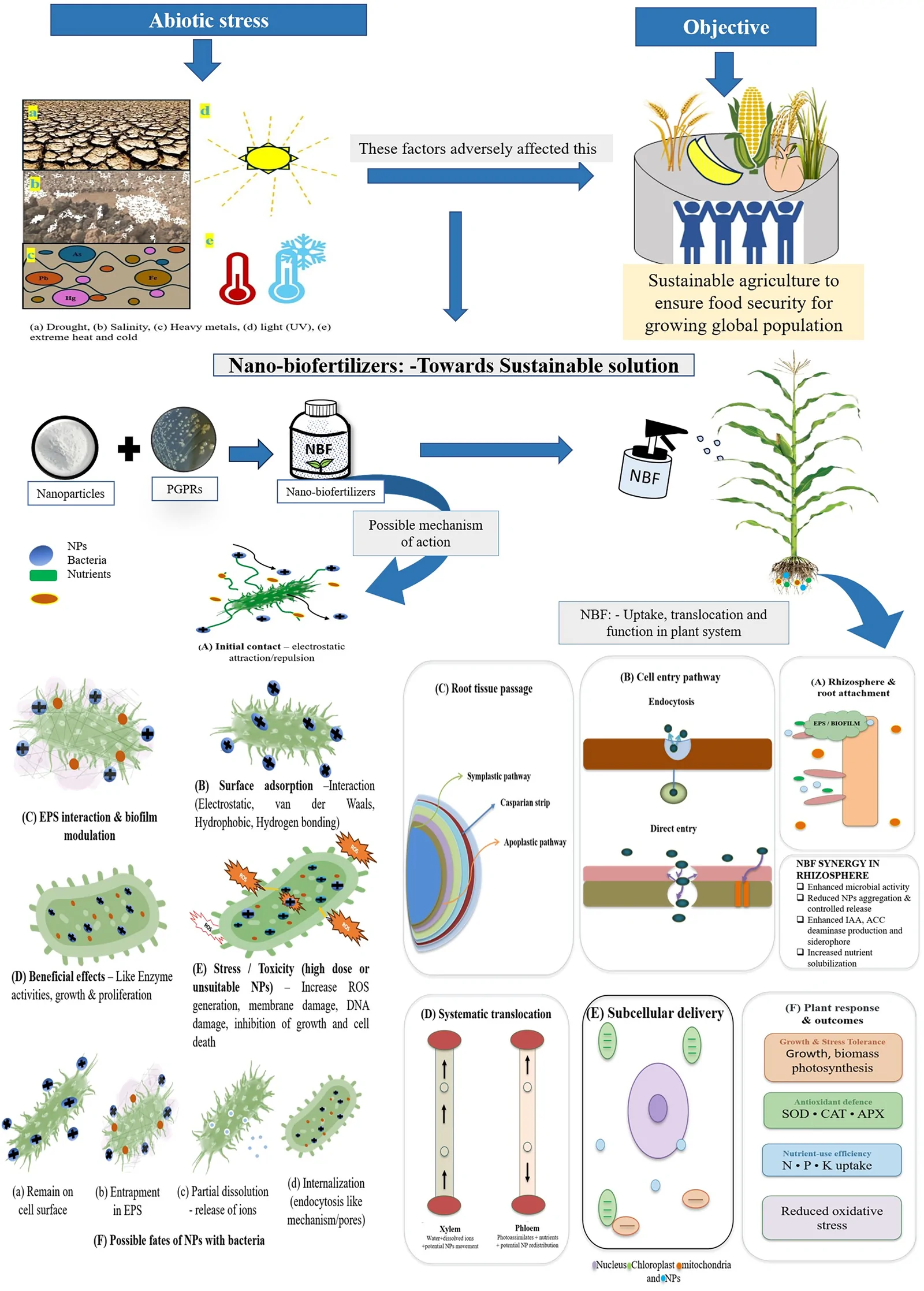

## Highlights

- Agriculture productivity is declined due to severe issues such as climate change, biotic and abiotic stress.
- Inorganic fertilizers have enormous capability but also raise the concern of soil degradation at the same time.
- Bio-fertilizer offers eco-friendly alternatives, but their limited yield remains constant
- Nanotechnology emerges as a promising tool to agricultural output but raises concerns over accumulation and potential health risks.
- Nano-biofertilizers are combination of potent beneficial microbes such as Plant growth promoting rhizobacteria (PGPR) and innovative nanoparticles.
- Nano-biofertilizers are highly efficient in controlled and targeted delivery at cellular level, highly absorbent property due to their specific size and reduces agricultural wastages.
- Nano-biofertilizers play significant role in enhancement of microbial permanence by escaping bacteria from heat, UV and adverse conditions (Nano-coatings), increased shelf life and active colonization.
- Nano-biofertilizers have enormous capability to enhance seed germination photosynthesis activity, chlorophyll content, biomass of plants, boost yield and abiotic and biotic stress relief.
- Nano-biofertilizers play a critical role in soil and environment health through climate mitigation, maximise water retention, nutrient and reduced dependency on inorganic fertilizers.

## Limitations

Despite of several advantages, nano-biofertilizers possess few limitations such as-

- Nano-biofertilizers are costly and complex as compared to other fertilizers available.
- The researches on process and execution of nano-biofertilizers are very less across the globe.
- High dose of nano-biofertilizers is capable of damaging the cell due to their penetrating abilities, ultimately stopping the growth of plant.
- There are no clear guidelines on uses of nano-biofertilizers.
- They can be used only after large scale field trials and nanotoxicity studies.

## 1 Introduction

The world’s population has been increasing and expected to reach 10 billion by 2050. This is raising the global concern for sustainable agriculture productivity and food security for massive populace (**Figure 1**) (Ahmed et al., 2023). On the other hand, agricultural production is steadily declining due to several biotic and abiotic stresses (Al-Mamun et al., 2021). Abiotic stress includes drought, salinity, heavy metal toxicity, light, excessive heat and cold (**Figure 1**) (Atkinson and Urwin, 2012). These factors are responsible for significant changes in the physiological and biochemical processes of plants (Khalid et al., 2019), which limit plant growth and productivity (Adeleke et al., 2022). Salt and drought stress primarily damage soil-microbes balance, leading to deterioration of soil quality (Singh et al., 2025) and accelerating hunger throughout the world (Zain et al., 2024). Approximately 2.3 billion individuals are vulnerable to food insecurity and deprived of enough safe and nutritious food (FAO, 2022). Hunger eradication is a key feature of the Sustainable Development Goal-2 (SDG-2). In order to fulfil current demand, agricultural output must be increased (Ranjan et al., 2021), which will eventually lead to excessive use of inorganic fertilizers (Meena et al., 2020). These inorganic fertilizers are capable of increasing crop yield but leave a chronic impression on the soil (Liu et al., 2014). Therefore, it is necessary to use environment-friendly techniques for sustainable agriculture such as organic farming (Azarbad, 2022) and nano-biofertilizers, a convincing and sustainable approach (Prasad et al., 2017).

**Figure 1:**
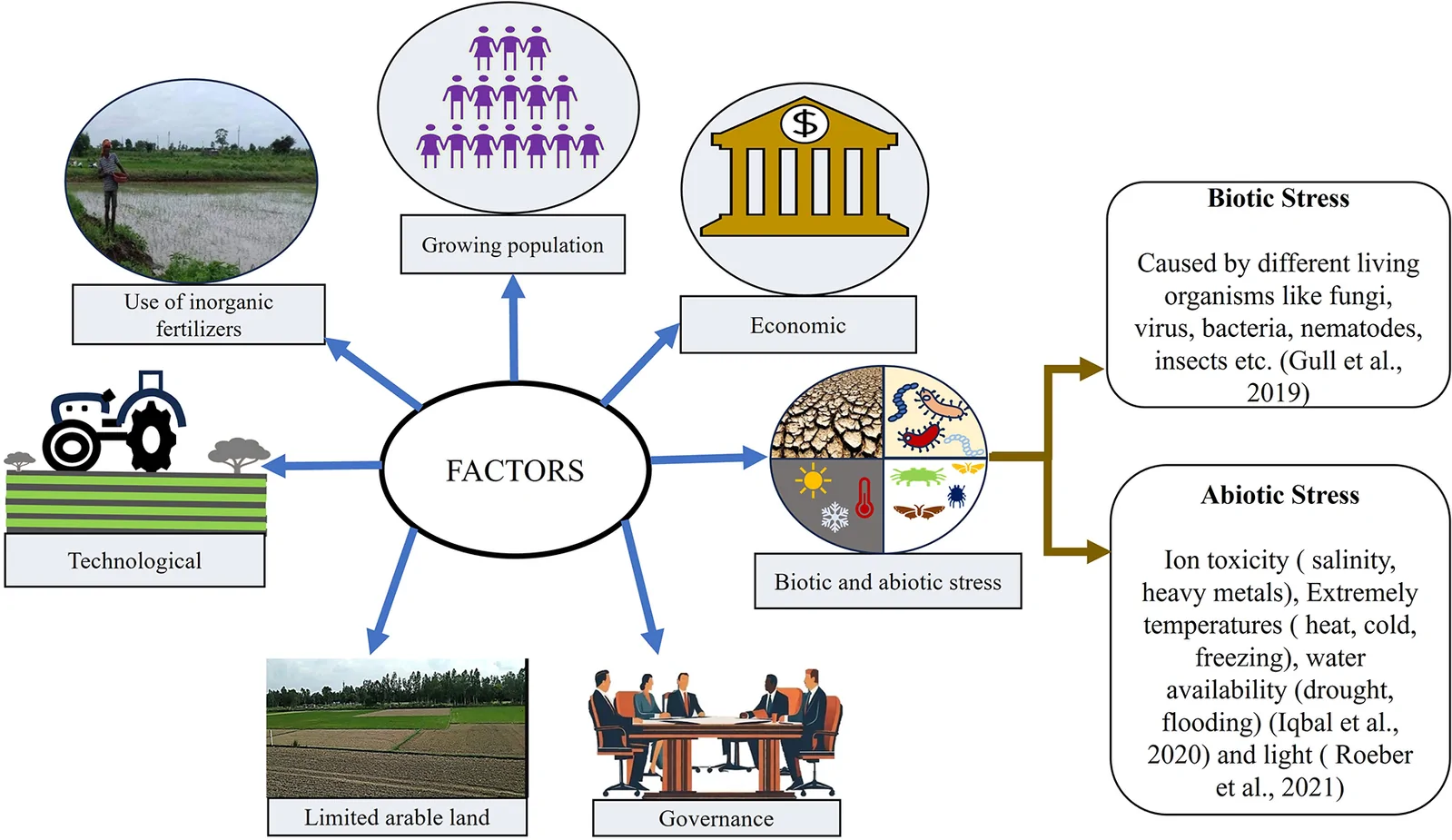
Factors affecting sustainable agriculture.

## 2 Methodology

The present study was conducted through a thorough literature survey regarding the concepts, aspects and various prospects of nano-biofertilizers. The data obtained from Google Scholar, Scopus and ResearchGate as primary sources of material. Keywords such as nanotechnology, stress physiology, organic farming, biofertilizers, PGPR, nano-fertilizers, nano-biofertilizer and combinations of nanoparticles and PGPR used as the major search terms. The collected data were thoroughly read and written in simple language. The data were also summarised in the form of tables and figures using MS Word and sketches. MS Office 16 version was used to write the manuscript.

## 3 Organic farming

The term “organic farming” refers to proactive and ecological management practices that reduces the risk to natural resources, human, animal health and biological variety (**Figure 2**) (Soni et al., 2022). Crop rotations and biological nitrogen fixation are two main pillars of organic farming responsible for enhancing soil fertility and nutrient cycle (Leifeld and Fuhrer, 2010; Seufert et al., 2017) physicochemical properties of soil, water holding capacity and soil’s microbial activity without having any adverse environmental impact (Vali et al., 2020).

**Figure 2:**
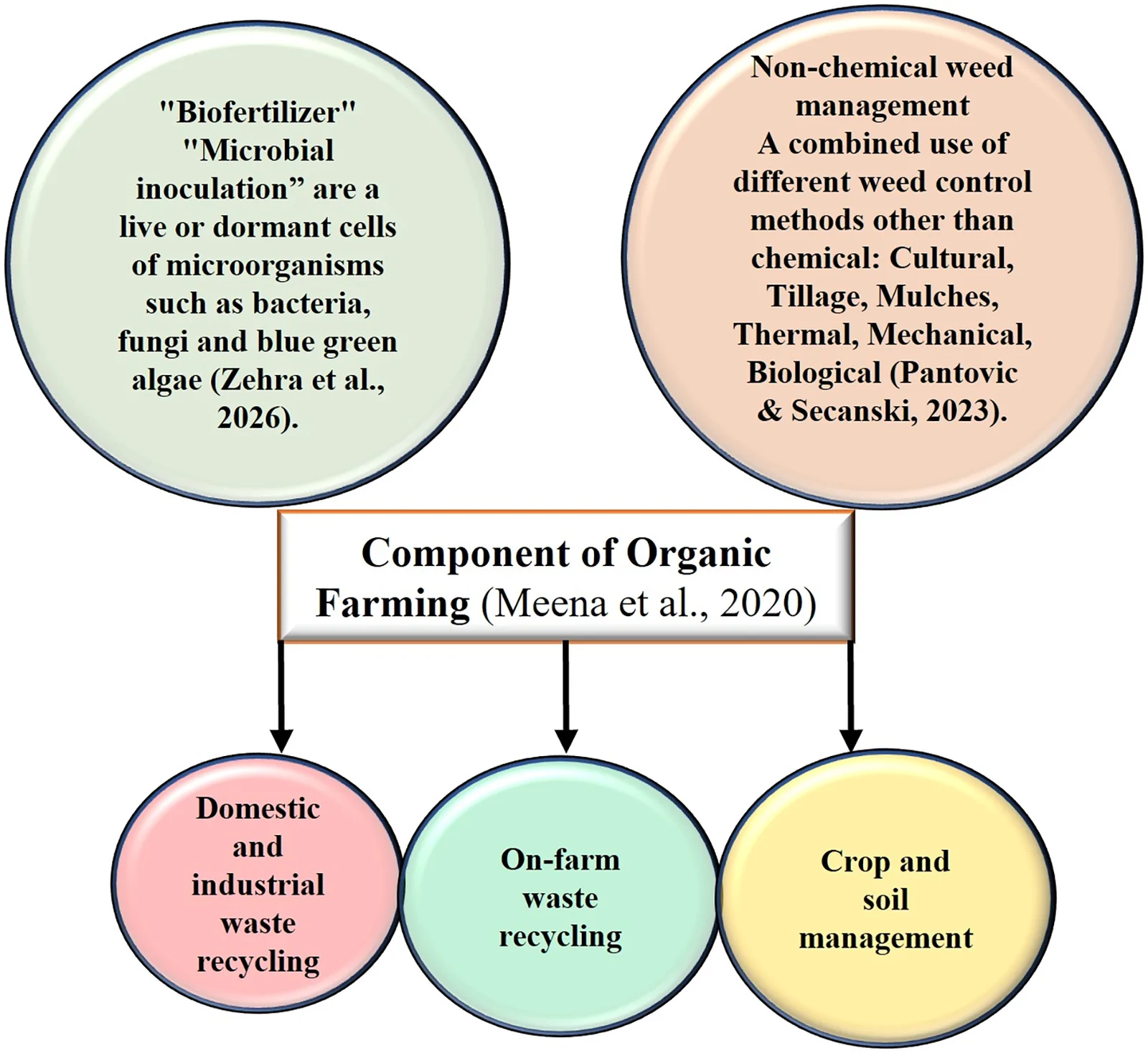
Systematic representation of the five pillars of organic farming. Collectively, these pillars define organic farming as a closed-loop system that substitutes synthetic fertilizers and herbicides with microbial and ecological alternatives.

### 3.1 Biofertilizers

Biofertilizers include soil bacteria that colonise the plant’s rhizosphere commonly known as plant growth promoting rhizobacteria (PGPRs) (Bhattacharjee and Dey, 2014). The rhizosphere is described as the soil volume that is directly attached to plant roots, root hairs and plant-derived substances (Tadrosova et al., 2025). It is generally divided into three regions, which are rhizoplane, endo and ecto rhizospheres (**Figure 3**) (Wijekoon and Weerasinghe, 2024).

**Figure 3:**
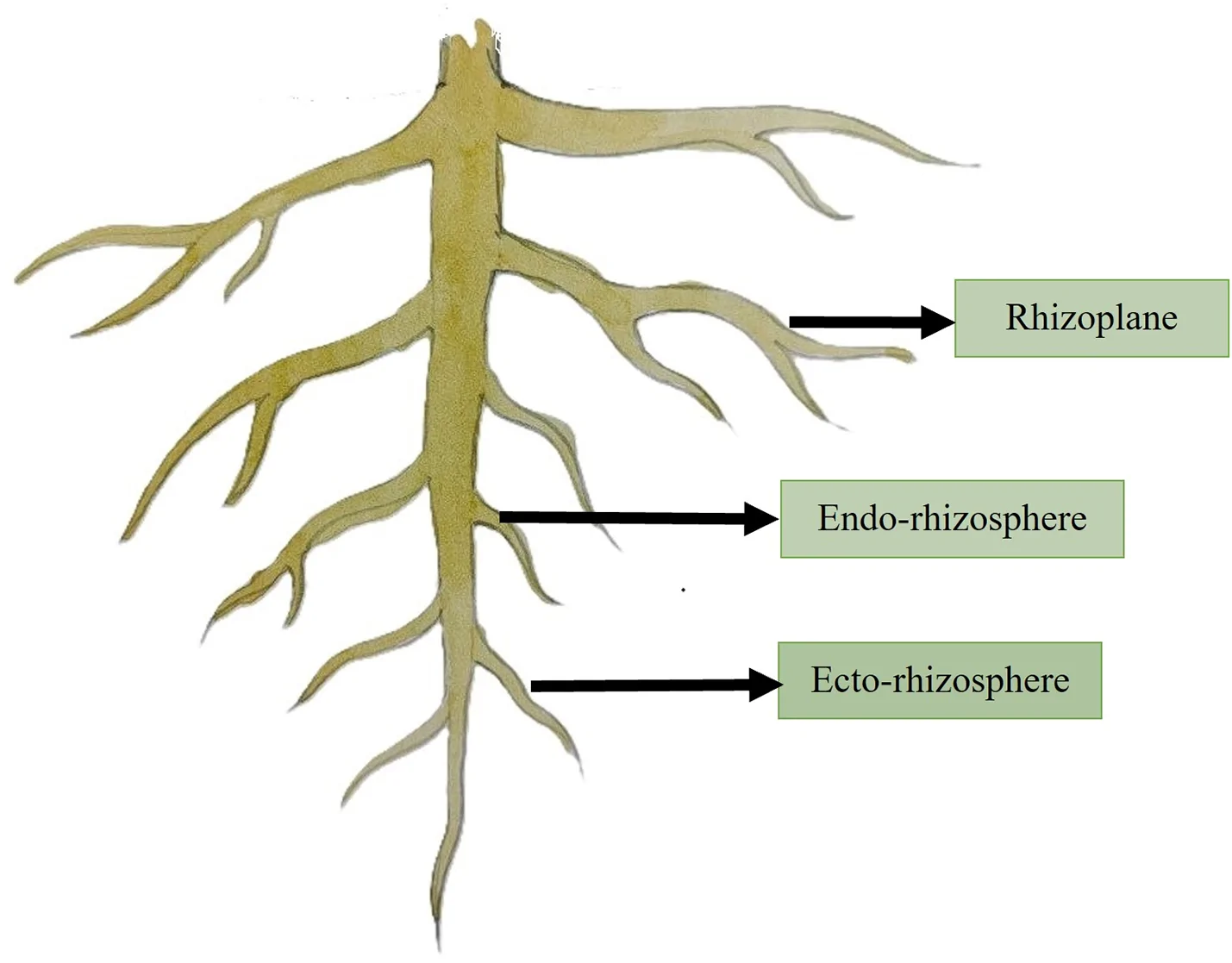
Different zones of rhizosphere.

PGPRs provide an effective way to mitigate soil and environmental degradation (AbdElgawad et al., 2023) and improve soil health (Verma et al., 2014; Yasin et al., 2016) by playing a critical role in soil fertility, plant nutrition and ecosystem resilience (Philippot et al., 2013). PGPRs are consortium of advantageous soil microorganisms that facilitate plant growth and development via plant-microbe interaction (Ma et al., 2019; Salwan et al., 2023). It has the capability to enhance plant development either through biological control of plant diseases or by direct action (Hartman and Tringe, 2019). PGPRs include two types of mechanism that is indirect action which occurs outside of the plant and direct action occur within the plant and directly influences the metabolism (**Figure 4**) (Pliego et al., 2011).

**Figure 4:**
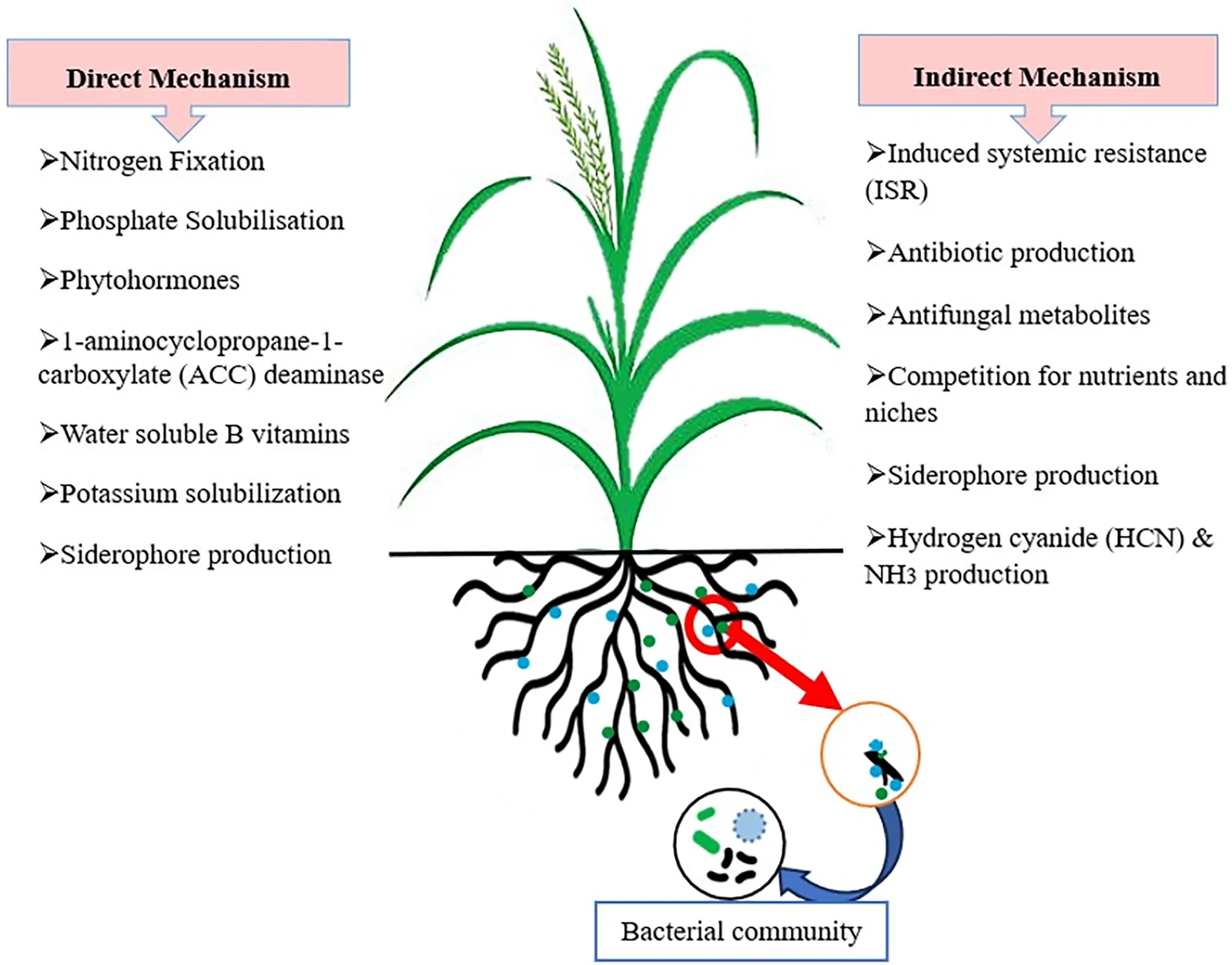
Systematic representation of dual mechanism (direct and indirect) of plant-growth promoting rhizobacteria (PGPRs).

PGPRs significantly enhanced nitrogen fixation in common bean (Garcia-Lopez et al., 2023). and maize plants (Kuan et al., 2016). The conversion of atmospheric nitrogen (N_2_) into ammonia (NH_3_) with the help of nitrogenase enzyme is known as biological N_2_ fixation (BNF) or diazotrophy (Lugtenberg et al., 2013). *Azospirillum brasilense* improved maize plant growth and yields (Ntsomboh-Ntsefong et al., 2025). It also regulates the level of phytohormones like cytokinin, indoleacetic acid (IAA), gibberellic acid (GA), ethylene, abscisic acid (ABA) and ACC deaminase enzymes (Egamberdieva et al., 2019). *Azospirillum* sp. and *Bradyrhizobium japonicum* species enhance the ABA, IAA and antioxidant capacity, which regulate plant physiology during drought, heavy metal and salinity stress in *Lactuca sativa* plants (Fasciglione et al., 2015; Seneviratne et al., 2016; Balkrishna et al., 2022). Additionally, biofertilizers enhance soil structure by promoting aggregation, aeration and increasing water retention capacity, which leads to overall soil health (Ghimirey et al., 2024). The genera of *Pseudomonas*, *Bacillus*, *Serratia*, *Rhodococcus*, and *Acinetobacter*, classified as plant growth-promoting rhizobacteria (PGPR), facilitate the solubilisation of ferric ions (Sahu and Sindhu, 2011; Rokhbakhsh-Zamin et al., 2011), inorganic phosphate into soluble forms (Sarmah and Sarma, 2023) and also improve the synthesis of antibiotics, antifungal, specifically 2, 4 – diacetylfluoroglucinol, pyrrolonitrin, hydrogen cyanide (HCN) and phenazine (Bhattacharyya and Jha, 2012). HCN plays a key role in the suppression of pathogenic bacteria and fungi (Nandi et al., 2017). PGPR strains, both alone and in consortia, induce systemic resistance (ISR) against pathogenic microorganisms (Mesele et al., 2026), e.g. black rot caused by *Xanthomonas campestris* pv (Liu et al., 2016).

Biofertilizers display two sides of the coin. It improves soil health and crop yields; on the other hand, having drawbacks such as low stability, sensitivity to adverse environmental conditions, requirement for high dosages over broad regions (Kumar et al., 2023) and shorter life span (Thirumurugan et al., 2024). There may be low crop productivity and nutritional shortage due to insufficient micro and macronutrient translocation (Carvajal-Munoz and Carmona-Garcia, 2012), resulting in the lack of ample food to feed the population (Roos et al., 2018).

## 4 Nano-fertilizers

To promote sustainability, there is a need to innovate traditional agricultural system by implementing eco-friendly approaches (Ranjan et al., 2021). In this regard nanotechnology has offered innovative possibilities for a sustainable future in the agricultural sector. It can serve as an alternative source of inorganic fertilizer because of its ability to minimise nutrient loss (through controlled release) and enhance solubility and stress resilience (**Table 1**) (Elizabath et al., 2019; Manjunatha et al., 2016). Nanoparticles (NPs) are synthesized via abiotic (physical/chemical) and biotic (biological/green) methods (Ndaba et al., 2022), using either a “top-down” or “bottom-up” approach (Abid et al., 2022). Nanoparticles made from biotic sources, i.e., plants and microbes, are more environmentally compatible compared to those from abiotic sources (Ditta et al., 2015). Reports have shown that ZnO NPs (Zinc oxide nanoparticle) improve plant growth (Shaik et al., 2020), enhance soil micronutrients, thereby improving soil health (Sharma et al., 2024), and increase photosynthetic activity in plants (Faizan et al., 2018); cerium oxide (CeO2) and titanium oxide (TiO2) nanoparticles also improve photosynthetic efficiency (Tighe-Neira et al., 2018). Nanoparticles, acts either as nutrients (micro or macro nutrients), transporters or supplements for nutrients (for example, by compositing with minerals) are known as nano-fertilizers (Kah et al., 2018). Nanomaterials have effectively improved agriculture but also raise concerns about accumulation and transmission across trophic levels. TiO2 nanoparticles smaller than 140 nm were found to accumulate in the roots of wheat (Larue et al., 2012). They also exhibit greater susceptibility to biomagnification in aquatic species after low-dose exposure (Kim et al., 2016). ZnO NPs (50 mg/L) induce oxidative stress and also accumulate in the roots and leaves of *Salvinia natans* (Hu et al., 2014). When crickets consumed zucchini plant leaves of CeO_2_ NPs exposed plant, they excreted faeces that contained cerium, which indicates that CeO_2_ NPs can be transferred from one trophic level to another (Hawthorne et al., 2014). On the other hand, it was also reported that the accumulation of iron oxide (Fe_3_O_4)_ nanoparticles was seen in the roots and leaves of the pumpkin plants (*Cucurbita maxima*) (Zhu et al., 2008). In *Arabidopsis thaliana* plants, the concentration of silver nanoparticles (AgNPs) increases from 100 to 150 mg/L, which increases the accumulation of NPs in the plant (Sosan et al., 2016). The effectiveness of nanoparticle is depended upon size, optimal dose and exposure time. Its size plays a critical role in surface area, reactivity and mobility. It has been reported that copper oxide NP size of 45nm shows increased nutrient accumulation, higher bioavailability, translocation and less oxidative stress as compared to large-size (510nm) in *Cucumis sativus* plant (Zong et al., 2022) and 25 nmNPs showing the highest H_2_O_2_ and MDA concentrations as compared to 250 nm NPs in *Glycine max* plant (Yusefi-Tanha et al., 2024). Increasing concentrations improve Cu ions in soil and uptake by plant, however optimal dose 100mg/kg stimulate growth and higher doses may induce stress in *Cucumis sativus* plants (Zong et al., 2022). Low dose of ZnO NPs (10mg/L) shows more efficiency as compare to large towards plant growth (Samaan et al., 2026). Ag nanoparticles with a 50-ppm concentration show optimum dose to promote growth of *Brassica juncea* plant by modulating their antioxidant status (Sharma et al., 2012). 100-ppm of sulphur NPs act as an optimal standard dose to get better productivity, cellular health, and reduce oxidative stress of *Mangifera indica* L. plant under biotic stress (powdery mildew) (Abou El-Nasr et al., 2025).

**Table 1:** Effect of nano-fertilizers on plant growth and development.

S.no.	Nano-fertilizer	Concentration/size	Exposer time	Environment condition	Observed effect	Reference
1	Carbon nanoparticles (CNPs)	1.0 ml/L	25 days	Controlled condition	Enhanced drought tolerance, better morphology, higher yield	(Vilas Boas et al., 2026)
2	Nano-hydroxyapatite (nHAP)	150 mg/kg 20-80nm	30 days	Greenhouse condition	Significantly boosting bulb size, yield, and nutrient uptake Effects comparable to conventional phosphorus fertilizer.	(Dwivedi et al., 2026)
3	Potassium–silica nanofluid	5 ml 8.6nm	35 days	Controlled condition	Boosted plant height significantly	(Rosanti et al., 2026)
4	CeO_2_ NPs	100 ppm	-–-	Controlled condition	Improves plants (*Oryza sativa L.*), growth under Chromium stress	(Sultan et al., 2026)
5	CaO NPs	50 µg/ml 30–50 nm	28 days	Greenhouse conditions	Improved photosynthetic efficiency and nutrient uptake in *Zea Mays L.* plants Improving ion mobility and promoting root development Reduced accumulation of Chromium in plants.	(AL-Huqail et al., 2024)
6	CaO NPs	50 mg/L 15–35 nm	20 days	Controlled condition	Improve plant growth and biomass of rice seedlings and also safe for cultivation of rice in fluoride contaminated field	(Koley and Mondal, 2024)
7	NPK + CeO2 NPs	200 ml (NPK) 12.1-36.8 nm 100 mg L^-1^ (CeO_2_ NPs) 16.6-25.5 nm	75 days	Open field condition	Head weight of cabbage increased three times to reach 1.88 Kg/plant as compare to control 0.51 Kg/plant Increased plant growth and photosynthesis activity.	(Abdulhameed et al., 2021)
8	ZnO NPs	100 ppm	146 days	Open field condition	Increased plant growth under drought stress in eggplant	(Semida et al., 2021)
9	Fe_3_O_4_ NPs	0.1 mg/L 20 nm	12 days	Greenhouse condition	Increased plant dry weights and antioxidants of the *Nicotiana benthamiana* plants Showed Fe accumulation in plant parts (Leaves, shoot and root). Increased plant disease-resistance ability	(Cai et al., 2020)
10	NPK Nano fertilizers based on chitosan nanoparticles	30–40 ppm 500nm	45 days	Greenhouse condition	Enhanced the nutrient uptake, chlorophyll content, photosynthesis process and growth of the coffee seedling	(Ha et al., 2019)
11	TiO_2_ -NPs	5–20 mg L^-1^ Average 30–35 nm	20 days	Hydroponic condition	• Improved the uptake of nutrients like N, P, K, Zn and Mn • Decreased the concentration of Fe and Cu minerals Increased concentration reduced chlorophyll content of leaves of Zea mays plant	(Daghan, 2018)
12	CeO_2_ NPs	100 mg/kg 10–30 nm (average 19 nm)	21 days	Controlled condition	Improved Photosynthesis rate and the Rubisco activity of soybean seedlings	(Cao et al., 2017)
13	Ag NPs	Lower concentration (0.05 mg ml^-1^and 0.1 mg ml^-1^) 20 nm	16 days	Controlled condition	Enhanced seedling of Zucchini plants	(Almutairi and Alharbi, 2015)
14	CuO NPs	500 mg dm^-3^	14 days	Controlled condition	• Increased H_2_O_2_ content, lipid peroxidation in root and reactive oxygen species generation of green peas plant Reduced plant growth	(Nair and Chung, 2015)
15	Au NPs	100–400 ppm 50 nm	10 days	Controlled condition	Increased length of shoot and root, antioxidative enzyme activities, H_2_O_2_ and proline contents of the *Brassica juncea*	(Gunjan et al., 2014)
16	ZnO NPs CeO_2_ NPs	400 mg/kg 10 nm 800 mg/kg 10 nm	53 days	Greenhouse condition	Positive impact on Starch, Globulin, Mg and Mn mineral. Negative impact of Cu and Mo mineral	(Zhao et al., 2014)
17	Mn NPs	0.05 mg/L 20 nm	Seed treated with nanoparticle solution for 4–6 hours	Controlled condition	Increased length of root and shoot and also fresh and dry matter of mung bean plants.	(Pradhan et al., 2013)
18	Ag NPs	60 ppm Average 20 nm	12 days	Greenhouse conditions	Improved stem and root elongation of the *Phaseolus vulgaris* and Zea mays plants	(Salama, 2012)

**Table 1** summarises the effects of nano-fertilisers on plant growth across different studies, with different concentrations ranging from 0.05 mg/L to 500 mg/kg, size 8.6 nm to 500 nm and exposure periods of 12 to 146 days in diverse environmental conditions. Observed effects include direct growth enhancement, altered nutrient profiles and metabolic or oxidative shifts. These effects are concentration and size-dependent, rather than uniformly beneficial.

## 5 Sustainable solution

A long-term solution is needed for sustainable agriculture and food security for a growing population. Nano-biofertilizers (NBFs) comprise of biofertilizers integrated with nanomaterials (Meena, 2025). NBFs can minimize problems like overuse of chemical fertilizers, slow action of biofertilizers and direct accumulation of nanoparticles by making PGPRs as a carrier (Jha et al., 2024; Shukla et al., 2013; Balachandar, 2012).

### 5.1 Nano-biofertilizers: new generation fertilizers

Nano-biofertilizers represent an innovative approach to improving agricultural productivity (Arora et al., 2024). NBFs work through multiple mechanisms, including controlled nutrient supply, improved microbial activity, and enhanced plant nutrient absorption (Al-Juthery et al., 2025). They improve soil structure and health in several ways, including restoring nutrient availability in the soil via phosphorus solubilisation and nitrogen fixation (Meena, 2025). Reports suggest that co-application of Fe-NPs (Iron nanoparticles) with *Bradyrhizobium japonicum* enhanced photosynthesis, nodulations and plant productivity in *Glycine max* L (Zehra et al., 2026). Nano-biofertilizers not only boost crop yield, nutritional value, shelf life and water conservation efficacy but also act as resistance-inducing agents to strengthen plant defences against pests and disease-causing organisms (Kumari and Singh, 2020). Iron oxide nanoparticles (dose 300 mg/kg) combined with *Bacillus firmus* improved seed germination by 45% as compared to alone (Benedetti et al., 2024).

## 6 Effect of nano-biofertilizers

### 6.1 Nitrogen

Nitrogen is the crucial element that plays an important role in the biochemical and physiological function of the plant (Ohyama, 2010; Leghari et al., 2016). Nano-biofertilizers have the ability to increase nitrogen fixation (Garg et al., 2023). With the combined application of nickel oxide (NiO) NPs and *Bacillus subtilis*, biological N_2_-fixation has been increased in soybean plants (Khan et al., 2025). Synergistic impact of *Bacillus megaterium* and zinc oxide nanoparticles can increase nitrite reductase activity in soil (Yan et al., 2020).

### 6.2 Phosphorus

Phosphorus is the second most crucial plant nutrient; hence, supplementation in adequate quantity is vital for plant growth and development (Safari et al., 2020). ZnO NPs (10ppm) and SiO_2_ NPs (1 ppm) applied together enhance the potential of *Bacillus megaterium* for phosphate solubilization (Zou et al., 2024), and SiO_2_ NPs (100 ppm) achieve the same effect for *Bacillus cereus-amazcala* (Ferrusquia-Jimenez et al., 2022). Interaction between Mycorrhizae, *Azotobacter chroococcum* and the potassium-based nano-fertilizer K_2_SO_4_ (1.5 g/L) increases phosphorus availability and macro-element absorption (Al-Fahdawi and Allawi, 2019). Using Fe_3_O_4_ NPs (100 ng/mL) with bacterial consortia increases phosphate solubilization (Kumar et al., 2020).

### 6.3 Potassium

Potassium is a vital macronutrient that plays an important role in plant growth, including membrane potential regulation, enzyme activation, protein synthesis, osmoregulation, sugar transport and stress adaptation (Xu et al., 2020; Johnson et al., 2022). More than 90% of the potassium available in the soil is insoluble (Prajapati and Modi, 2012). Therefore, plants require essential macronutrients for proper growth (Sindhu et al., 2016). Hence, when nanoparticles are integrated with potassium-solubilising microorganisms, it offers decomposition of insoluble potassium into soluble potassium for plant uptake (Pandey and Saharan, 2025). The combination of selenium NPs with PGPRs such as *Pseudomonas fluorescens* and *Bacillus cereus* increased the potassium content in the leaves of foxtail millet (Nasibi et al., 2022).

### 6.4 Indole-3-acetic acid

IAA significantly regulates plant development processes, differentiation and leaf abscission. *Pseudomonas aeruginosa* combined with chitosan and gold nanoparticles (AuNPs) has shown increased production of IAA, 7.16 ± 0.28 µg/mL and 9.56 ± 0.28 µg/mL respectively, compared to *Pseudomonas aeruginosa* alone, 5.33 ± 0.28 µg/mL (Panichikkal and Krishnankutty, 2022). AuNPs also enhanced the IAA-producing potential of *Pseudomonas monteilii* (Panichikkal et al., 2019). *Streptomyces* bacteria in combination with potassium nanoparticles increased IAA production in *Calendula officinalis L* (Sarlak et al., 2024). *Bacillus megaterium* with ZnO-NPs and SiO2-NPs at lower concentrations improved IAA synthesis (Zou et al., 2024).

### 6.5 pH of the soil

The pH is the primary soil characteristic that determines diversity, biomass and microbial composition. Excessively high or low pH level affect the metabolic processes of bacteria (Naz et al., 2022) and also adversely affect the nodulation process between microorganisms and leguminous plants (Jachak, 2017). pH influences the solubility of nutrients in the soil and their availability (Hussain et al., 2026) and the growth of the plants (Joshi et al., 2026). PGPRs can influence the pH of the rhizosphere (Ipek and Esitken, 2017). The pH dropped dramatically from 8.15 to 8.01 when PGPRs and SiNP was applied together (Alharbi et al., 2022). This shows the synergistic application of PGPR and nanoparticles has the potential to influence pH of soil and promote plant growth.

### 6.6 Soil health

The excessive use of inorganic fertilizer in agriculture reduces soil fertility by altering its essential physicochemical properties including pH, organic matter, potassium, phosphorus, nitrogen, and electrical connectivity etc (Fazelian and Yousefzadi, 2022). Nano-biofertilizers have the potential to improve soil fertility and slow down soil deterioration by retaining nutrients, water, and soil structure. The synergistic implementation of NPs and PGPRs has shown an effective strategy to enhance soil health (Rajput et al., 2024), the population of advantageous microbiota (Chaudhary et al., 2022) and nutritional status by phosphorus solubilization and nitrogen fixation (Mala et al., 2017; Upadhayay et al., 2023). Synergistic effect of *Bacillus* spp. and nano-zeolite significantly increases organic carbon (OC) content of soil (Khati et al., 2018). Combined effect of *Nocardiopsis* sp. strain JM18 and Selenium Nanoparticles (SeNP) improved physio-chemical properties of soil (Verma et al., 2014).

## 7 Effect of nano-biofertilizers on abiotic stress

### 7.1 Drought

Drought and its derivative stress conditions are one of the significant factors that negatively affect crop production. Nano-biofertilizers reflect great potential to mitigate adverse effects of drought and also enhance nutrient availability, improved soil quality, essential oil yields and overall promote plant growth (**Table 2**) (Eskandari et al., 2023). Together, ZnO NPs and *Pseudomonas* sp. improved the growth of wheat plants under heat and drought stress (Azmat et al., 2022). Synergistically SiO_2_ NPs (size 45.96 – 48.73 nm) and *Azospirillum brasilense* increased induced systemic resistance (ISR) in *Triticum aestivum* under drought stress (Akhtar et al., 2021a). Also, ZnO NPs (size <100 nm) with *Azospirillum brasilense* effectively improved the grain quality and antioxidant enzymes of wheat plants under drought stress (Muhammad et al., 2022).

**Table 2:** Combined effect of nanoparticles and biofertilizers on plant growth and development under abiotic stress.

S. no.	Nanoparticle + PGPR	Concentration/size/applied/synthesis of nanoparticles	Environment conditions/exposer time	Stress/plant	Effect	Reference
1	ZnFe2O4 NPs + *Alcaligenes pakistanensis*	100 mg/L 25 ± 5 nm	Greenhouse condition	Drought/*Phaseolus vulgaris L.*	• Significantly improves germination, vegetative growth and anatomical traits (stomata, guard cells and trichomes)	(Nafees et al., 2026)
2	SiNPs + *Bacillus cereus*	150mg Less than 100 nm Foliar spray Physical method (Hand grinding)	Open field condition November 2023 to April 2024 and November 2024 to April 2025	Rain-fed condition/*Triticum aestivum L.*	• Increased 18.10% of shoot height, 97.43% of germination index, 22.54% of total chlorophyll, 12.94% of relative water content, 18.75% of phenolic content, 26.93% of biological yield and 22.83% of catalase activity as compare to control	(Umer et al., 2026)
3	AuNPs + *Stenotrophomonas rhizophila*	100 µg m/L 78.15 ± 10.89 nm Green synthesized	Greenhouse condition	*Solanum lycopersicum*	• Improved disease suppression and growth of plants	(Ali et al., 2026)
4	Chitosan nanoparticles + *Trichoderma harzianum*	100 ppm Foliar spray Ionic gelation method	Open field condition Winter seasons of 2021 and 2022	Arid region/Garlic plants	• Increased nutrient (NPK), 16% height, 25% dry weight, 15% bulb diameter, photosynthetic pigment of plant	(Shalaby, 2025)
5	ZnO-NPs + *Bacillus* sp.	Average 30 ± 5 nm	Greenhouse condition	Cucumber	• Improved Antibacterial, antioxidant and plant growth-promoting properties	(Helaly et al., 2025)
6	ZnO-NPs + PGPR (Nitrogen fixing, phosphorous solubilizing and potassium bacteria with *Rhizobium* strain TAL-169)	100 mg/L 25–50 nm Foliar spray Sol-gel method	Greenhouse condition 160 days	Cadmium/*Triticum aestivum L.*	• Increased antioxidative enzymatic activity • Increased productivity, growth and photosynthetic activity • Lower the rate of *TaEIL1* gene expression • Level of Cd decreased in plant	(Sehrish et al., 2024)
7	SiO2-NPs + AMF (Arbuscular mycorrhizal fungi) and PGPR (*Glomus mosseae*, *Pseudomonas putida* strain 18,798, *Flavobacterium*)	60 mg/L 20–30 nm	Greenhouse condition	Salinity/*Triticum aestivum L.*	• Improved photosynthesis, chlorophyll and carotenoid content, plant growth • Improved the level of Si, P, and K+ • Decreased Na+ uptake	(Ahmadi-Nouraldinvand et al., 2024)
8	Si-NPs + Bio-fertilizers (*Flavobacterium*, *Pseudomonas putida* strain 18,798 and *Glomus mosseae*)	60 mg/L 20–30 nm Foliar spray	Greenhouse condition	Salinity/*Triticum aestivum L.*	• Enhanced biochemical and physiological mechanisms	(Ahmadi-Nouraldinvand et al., 2023)
9	TiO 2 -NPs + *Bacillus mycoides* PM35	50 μg/ml	Greenhouse condition Seed priming 24 hours	Cadmium/*Hordeum vulgare L.*	• Enhanced the vital nutrient in plant • Reduced the level of cadmium in plants	(Ma et al., 2023)
10	ZnO NPs + PGPR (*Bacillus subtilis* MF497446, *Bacillus circulance* NCAIM B.02324, *Bacillus coagulans* NCAIM B.01123 and *Azospirillum lipoferum* SP2)	500 mg/L 30–70 nm Foliar spray	Open field condition 80 days	Sodic-saline/ *Triticum aestivum L.*	• Increased relative water content, chlorophyll (a and b), uptake on nitrogen (57%), grain yield, and Zn (grain 117% and straw 72%) • Reduced Hydrogen Peroxide (H_2_O_2_) and Malondialdehyde (MDA) content • Increased superoxide dismutase (SOD), catalase (CAT) and peroxidase (POD)	(Alharbi et al., 2023)
11	CS-NPS (chitosan nanoparticles) + Biofertilizers (*Pantoea agglomerans* strain O4, *Pseudomonas vancouverensis* strain S19 and *Thiobacillus* sulfur-oxidizing bacteria)	200 nm Ionotropic gelation methods	Open field condition	Drought/ *Camelina sativa L.*	• Increased the chlorophyll, carotenoid and proline content • Reduced Malondialdehyde (MDA) and Hydrogen Peroxide content	(Haghaninia et al., 2023)
12	CuO NPs + *Trichoderma harzianum*	50 nm Green synthesised	Controlled condition	Salinity/ *Arabidopsis thaliana.*	• Reduction of oxidative stresses including ROS, H_2_O_2,_ and MDA • Enhancement of antioxidant enzymatic activities – SOD and CAT • Improved vegetative growth total chlorophyll contents	(Shah et al., 2023)
13	ZnO-NPs + PGPR	50–100 mg/L 20–30 nm Foliar spray	Greenhouse condition	Arsenic/*Zea Mays L.*	• Enhancement in relative water content (RWC) 43%–50% and plant biomass • Reduction in MDA content 46%–57% • Improvement of Proline, total protein, chlorophyll (a, b), carotenoids and total soluble sugars content.	(Khan et al., 2022)
14	MgO NP + EMs (effective microorganisms)	50 or 100 µg m/l Average size less than 100 nm Foliar spray	Open filed condition	Salinity/*Ipomoea batatas L.*	• Enhanced the antioxidant activity • Improved RWC, MSI (cell membrane integrity)	(Abd El-Mageed et al., 2022)
15	Mo NPs + *Bacillus* sp. strain ZH16	100 mg/L 24.5–57.6 nm Seed priming Biological synthesis	Greenhouse condition 30 days	Arsenic/*Triticum aestivum L*.	• Improved morpho-physiological parameters • Increase uptake of K^+^ the availability of Na^+^ and Ca^2+^ ions	(Ahmed et al., 2022)
16	Si-ZnNPs + PGPMs (*Bradyrhizobium japonicum* (USDA 110) and *Trichoderma harzianum*)	Si – 198nm Zn <100 nm 500mg/L (250mg/L + 250 mg/L) Foliar spray	Open field condition 60 days	Water stress and soil salinity/*Glycine max*	• Improved seed quality (i.e., protein and carbohydrate content) • Reduced the effect of water and salinity stress	(Osman et al., 2021)
17	TiO2 NPs + *Pseudomonas fluorescens*	500 mg/kg 15–40 nm Mixed with soil Hydrolysis or precipitation methods	Greenhouse condition 80 days	Cadmium (Cd)/*Trifolium repens*	• Promote phytoremediation • Enhanced plant biomass	(Zand et al., 2020)
18	CuO NPs + *Pseudomonas chlororaphis* O6	300mg/kg Mixed with soil	Controlled condition 6 Days	Drought/ *Triticum aestivum L.*	• Enhanced lignification in stem cells • Enhanced the level of nitric oxide in root of the wheat plant	(Jacobson et al., 2018)

### 7.2 Salinity

Salinity occurs due to increasing salt content in the soil (Nigam et al., 2026). In saline conditions, chloride and sodium ion accumulate in plant tissue, affecting ion homeostasis, nutrient uptake (Fragoso-Iniguez et al., 2025), decreasing the degree of opening and closing of stomata (Trusca et al., 2026) and also, disrupting physiological processes by inducing osmotic and ionic stress (Yadav et al., 2020). In plants, water deficiency combined with osmotic stress due to excess salts, which inhibits cell expansion and overall plant development (Naz et al., 2021). Silver nanoparticles (dose 5 ppm) in combination with *Pseudomonas moraviensis* effectively increased onion bulb weight in salinity (Jahangir et al., 2020). Synergistically ZnO NPs (dose 17 mg/L) and biofertilizer enhances the growth of Safflower (*Carthamus tinctorius* L.) plants in terms of fresh and dry weight and length of shoot and root under salinity (Yasmin et al., 2021). When ZnO (dose 50 mg/L) nanoparticle inoculated with *Bacillus subtilis*, they increased the leaf area index, weight and length of shoot and root of wheat plants under salt stress (Ayman et al., 2024). The combined application of nano urea and PGPR enhance plant growth of *Amaranthus tricolor* under salt stress (**Figure 5**) (Elbagory et al., 2025).

**Figure 5:**
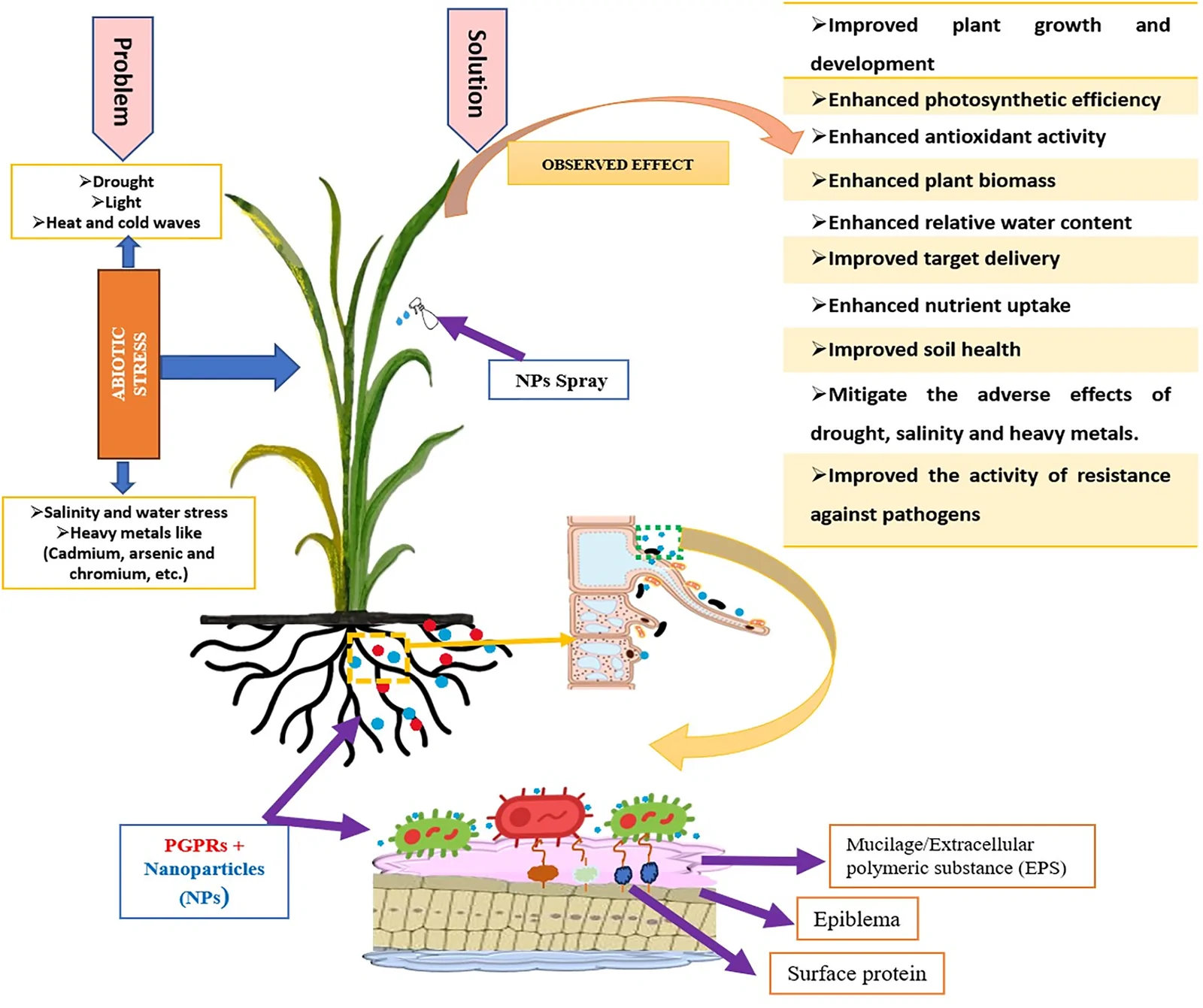
Systematic representation of the effect of nano biofertilizerson plant growth and development to mitigate abiotic stress.

### 7.3 Heavy metal

Heavy metals (HMs) exhibit heightened toxicity to both flora and fauna. Higher concentration of metal in the soil affects the physiological, metabolic, genetic and seed germination capabilities of the plant system (Yadav and Kumar, 2026; Bharti and Sharma, 2022). Heavy metals such as arsenic and cadmium induce the synthesis of reactive oxygen in plants (Yasin et al., 2018; Varallyay et al., 2015; Nurchi et al., 2020) which causes oxidative stress (Rao et al., 2025). When zinc oxide nanoparticles (size 30–70 nm) with bacteria *(Bacillus cereus and Lysinibacillus macrolides)* act synergistically, they enhance plant growth and reduce heavy metal stress (Akhtar et al., 2021b). *Bacillus subtilis* combined with iron oxide nanoparticle (dose 15 mg/L) increase synthesis of stress-tolerance polyamines like putrescine in *Cucurbita moschata* plants under arsenic stress (Mushtaq et al., 2020). And also, ZnO-NPs with *Bacillus fortis* IAGS 223 significantly enhanced growth of *Cucumis melo* seedlings under cadmium stress (**Figure 5**) (Shah et al., 2021).

**Table 2** Illustrate various types of nano-fertilizers and biofertilizers with different nanoparticle sizes and concentrations across diverse plants, including cereals and vegetables etc. under different abiotic conditions such as salinity, drought, and heavy-metal stress. Observed effect shows that combine effect can mitigate stress.

## 8 Possible mechanism

The mechanism of synergetic effect of nanoparticles with soil microbes involves direct interaction. The properties of nanoparticles (NPs) are ascribed to their small size of 1–100 nm, which enhances specific surface area and exhibits high surface reactivity (Ivask et al., 2014). Nanoparticles can bind to the upper receptors of plant cell (Chauhan et al., 2026) and microbial cell due to their surface sensitivity. Usually NPs applied through foliar spraying, mix with soil and seed priming after which it can be absorbed or moved throughout plant cell (Ahmadi-Nouraldinvand et al., 2026). The activities of various nanoparticles vary with their respective size. A few reports support the binding efficacy of gold nanoparticles of 45 nm with *P. fluorescence, B. subtilis* and *P. elgii* and encourage their luxurious growth; in contrast, disrupting the cells of *P. putida* resulted in no growth (Shukla et al., 2015). AuNPs were found accumulate in the seedlings of *B. juncea* when directly applied and this has helped the seedlings to expand the average root length to 11.62 cm at a 100-ppm concentration of AuNPs (Gunjan et al., 2014).

## 9 Future blueprint

Nano-biofertilizers are capable of providing a potential blueprint for modern and sustainable agriculture. This involves nutrient management through the development of stable nano-bio formulations and nano-encapsulated, carrier-based system. It also involves nanoparticle–microbe integration for specific crops, soil conditions, and nutrient deficiencies to enable targeted application. Further, nano-biofertilizers enhance microbial diversity, crop health and soil quality (Kumari and Singh, 2020). Their efficiency is dependent particularly on the size and dose of nanoparticles, ultimately necessitating a threshold for optimal utilisation of nano-biofertilizers (Zong et al., 2022). Overall, this approach promotes efficient resource use, reduces environmental risks, and supports climate-smart and sustainable agriculture. Furthermore, multi-location and long-term trials will be required to establish reliable application rates, standardise protocols, and identify doses maximising agronomic benefits and minimising phytotoxicity and environmental risks. Finally, successful adoption will require clear regulatory frameworks, cost-effective production, farmer-oriented training, and effective dissemination strategies. Together, these measures can support the development of nano-biofertilizers that improve nutrient-use efficiency, reduce environmental impacts and contribute to climate-smart, sustainable agriculture.

## 10 Conclusion

A paradigm shift toward sustainable agriculture by the use of soil microorganisms and nanomaterials. This strategy holds great potential for reducing negative environmental impact while satisfying food demand of the population. It offers a great opportunity towards sustainable agriculture by addressing the limitations of inorganic fertilisers, adverse impact of abiotic stress, organic farming and nanotechnology. The positive outcomes of nano-biofertilizers include improving the physio-chemical properties of soil, phytoremediation, reduction of oxidative stresses including ROS, H_2_O_2,_ and MDA, photosynthetic activity, target delivery, and antioxidant activities like SOD, CAT in plants. Therefore, it is vital to embrace the advance research in this area in order to shape the mechanism between nanoparticle and biofertilizers - initial contact and try to explain, the attachment of nanoparticle on bacterial cell and the mechanism of action thereafter.

Further, advancement of research to evaluate the benefits and risks associated with nano-biofertilizers, particularly regarding their impact on soil health, plant physiology, microbial communities, nanoparticle uptake and translocations trophic level (Prasad et al., 2017; Sambangi et al., 2022). Comprehensive field-based studies are also needed to assess their long-term environmental effects and viability across diverse agricultural conditions (Verma et al., 2022). Therefore, multidisciplinary research is required to overcome the gap in the link between nanoparticles and biofertilizers for responsible application in sustainable agriculture.
